# Effectiveness of Dance-Based Interventions on Depression for Persons With MCI and Dementia: A Systematic Review and Meta-Analysis

**DOI:** 10.3389/fpsyg.2021.709208

**Published:** 2022-01-05

**Authors:** Ying Wang, Mandong Liu, Youyou Tan, Zhixiao Dong, Jing Wu, Huan Cui, Dianjun Shen, Iris Chi

**Affiliations:** ^1^School of Philosophy and Sociology, Lanzhou University, Lanzhou, China; ^2^Research Institute of Social Development, Southwestern University of Finance and Economics, Chengdu, China; ^3^Shanghai Lixin University of Accounting and Finance, Shanghai, China; ^4^USC Suzanne Dworak-Peck School of Social Work, University of Southern California, Los Angeles, CA, United States; ^5^Edward R. Roybal Institute on Aging, University of Southern California, Los Angeles, CA, United States

**Keywords:** depression, anxiety, meta-analysis, dance-based intervention, GRADE, MCI, dementia

## Abstract

**Background:** There is a growing need to offer appropriate services to persons with mild cognitive impairment (MCI) and dementia who are faced with depression and anxiety distresses beyond traditional pharmacological treatment. Dance-based interventions as multi-dimensional interventions address persons' physical, emotional, social, and spiritual aspects of well-being. However, no meta-analysis of randomized controlled treatment trials (RCTs) has examined the effectiveness of dance-based interventions on depression and anxiety among persons with MCI and dementia, and the results of RCTs are inconsistent. The study aimed to examine the effectiveness of dance-based interventions on depression (a primary outcome) and anxiety (a secondary outcome) among persons with MCI and dementia.

**Methods:** A systematic review with meta-analysis was conducted. The inclusion criteria were: population: people of all ages with MCI and dementia; intervention: dance-based interventions; control group: no treatment, usual care, or waiting list group; outcome: depression and anxiety; study design: published or unpublished RCTs. Seven electronic databases (Cochrane, PsycINFO, Web of Science, PubMed, EBSCO, CNKI, WanFang) were searched from 1970 to March 2021. Grey literature and reference lists from relevant articles were also searched and reviewed. The Cochrane “Risk of Bias” tool was used to assess study quality. RevMan 5.4 was used for meta-analysis and heterogeneity was investigated by subgroup and sensitivity analysis. GRADE was applied to assess the evidence quality of depression and anxiety outcomes.

**Results:** Five randomized controlled trials were identified. Sample sizes ranged from 21 to 204. The risk of bias was low, except for being rated as high or unclear for most included studies in two domains: allocation concealment, blinding participants and personnel. Meta-analysis of depression outcome showed no heterogeneity (*I*^2^ = 0%), indicating that the variation in study outcomes did not influence the interpretation of results. There were significant differences in decreasing depression in favor of dance-based interventions compared with controls [SMD = −0.42, 95% CI (−0.60, −0.23), *p* < 0.0001] with a small effect size (Cohen's *d* = 0.3669); Compared with the post-intervention data, the follow-up data indicated diminishing effects (Cohen's *d* = 0.1355). Dance-based interventions were more effective in reducing depression for persons with dementia than with those having MCI, and were more effective with the delivery frequency of 1 h twice a week than 35 min 2–3 times a week. Also, one included RCT study showed no significant benefit on anxiety rating scores, which demonstrated small effect sizes at 6 weeks and 12 weeks (Cohen's *d* = 0.1378, 0.1675, respectively). GRADE analysis indicated the evidence quality of depression was moderate, and the evidence quality of anxiety was low.

**Conclusions:** Dance-based interventions are beneficial to alleviate depression among persons with MCI and dementia. More trials of high quality, large sample sizes are needed to gain more profound insight into dance-based interventions, such as their effects of alleviating anxiety, and the best approaches to perform dance-based interventions.

## Introduction

The importance of treating and researching MCI and dementia has gained widespread recognition in recent decades. About 16% of older adults suffer from MCI (World Health Organization, 2017a), and ~50 million people worldwide are affected by dementia (World Health Organization, 2020). Additionally, there will be 65.7 million people with dementia by 2030 (Alzheimer's Association, 2019), and 152 million by 2050 (World Health Organization, 2017b). Different stages and different symptoms of MCI and dementia impair one's cognition, thinking, and memory, and lead to social functioning decline, problematic behaviors, negative emotions, psychological symptoms, such as depression (Karkou and Meekums, 2017; Renom et al., 2020), and anxiety (Shaji et al., 2009; Krishnamoorthy and Anderson, 2011).

Depression and anxiety are among behavioral and psychological symptoms of dementia (BPSD) which are strongly correlate with and prevalent and persistent during the course of the progression of cognitive impairment (Cerejeira et al., 2012; van der Linde et al., 2016; Zhao et al., 2016). Depression is a highly prevalent concomitant of dementia (Grammes and Kubiak, 2020), causing distress, reducing the quality of life, exacerbating cognitive and functional impairment, and increasing caregiver stress. Even mild levels of depression can significantly add to the functional impairment of persons with MCI and dementia, while the severity of psychopathological and neurological impairments increases with that of depression (Gutzmann and Qazi, 2015). Although it is still under debate whether depression leads to dementia or depression is one of the symptoms of dementia (Kuo et al., 2020; Yang et al., 2021), there is no doubt that depression is closely related to MCI and dementia. Thus, identifying and alleviating depression for persons with MCI and dementia is an important strategy to facilitate the prevention and treatment of MCI and dementia. Also, the prevalence of anxiety in persons with MCI and dementia ranges from 8 to 71%, resulting in poor outcomes (Kwak et al., 2017). Persons with MCI and dementia with co-morbid anxiety have more difficulties with activities of daily living, leading to greater functional impairment and resulting in early long-term care (Cummings et al., 2015; Brown Wilson et al., 2019). It is also associated with having poor quality of life (Livingston et al., 2014; Kwak et al., 2017), problematic behaviors, nighttime awakenings, worse neuropsychological performance (Seignourel et al., 2008), and increased mortality (Tatsumi et al., 2009). Therefore, evaluating and treating depression and anxiety among persons with MCI and dementia is urgent (Grammes and Kubiak, 2020). More mental health services should be used to help persons with MCI and dementia deal with their depression and anxiety.

Traditional pharmacologic treatments are used to relieve depression or anxiety among persons with MCI and dementia (Dafsari and Jessen, 2020). Besides, there are many non-pharmacologic interventions, such as physical exercise (Aliev et al., 2013), mindfulness-based cognitive therapies (Aguirre et al., 2017), and music interventions (Ing-Randolph et al., 2015). The literature highlights that given the complexity of MCI and dementia' symptoms, researchers and practitioners should take a multi-dimensional and comprehensive perspective (Karkou and Meekums, 2017). Using diverse treatments (e.g., non-pharmacological interventions) and multi-component approaches might be most effective (Bennett et al., 2021). Non-pharmacologic interventions have been shown to effectively manage neuropsychiatric symptoms from comprehensive perspectives, including cognitive, emotional, physical, social aspects, and thus should be considered first before starting pharmacological treatments (Brasure et al., 2016; Austrom et al., 2018).

Dance-based interventions are any type of movement-with-music activity, such as tango, waltz, ballroom, polka, jazz, foxtrot, cha-cha, rumba, samba, bolero, and salsa. Dance-based interventions focused on dynamic balances of the physical movements with music's rhyme and rhythm (e.g., yoga and meditation focus on the posture of the static body, and thus they do not belong to dance-based interventions) (Ucznik and Loesche, 2017; Leach and Stevens, 2019). Dance-based interventions as non-pharmacologic interventions have gained rising attention and recognition (Burns, 2009; Hayes and Povey, 2010; Kiepe et al., 2012; Karkou and Meekums, 2017), although dance has been part of many cultures and histories for ages. The National Institute for Health and Care Excellence (NICE) in England recommends dancing as an intervention for dementia (Lyons et al., 2018). Literature shows that dance-based interventions as a mind-body movement can improve the cognitive function among aging adults (Wu et al., 2019), or persons with MCI (Chan et al., 2020; Hewston et al., 2020), and dementia (Lyons et al., 2018; Mabire et al., 2018). Regarding global cognition, dance-based interventions are more effective than physical exercises among persons with MCI, and more effective than combined training of cognitive and physical exercise among persons with cognitive impairments (Wang et al., 2018). Dance-based interventions can reduce psychological distress in patients with depression (Kiepe et al., 2012) and anxiety, increase quality of life, interpersonal and cognitive skills (Koch et al., 2019). Literature also highlights the advantages of dance-based interventions which include multidimensional components (Bruyneel, 2019), showing that it could effectively deal with the complexity of symptoms (Karkou and Meekums, 2017) and with no adverse effects (Bruyneel, 2019). They can also relieve depression and anxiety for persons with neurocognitive disorders from physical, psychological and other dimensions by adopting mind-body movement (Bruyneel, 2019; Wu et al., 2019). Dance-based interventions are recognized as more integrated interventions (Lyons et al., 2018) and holistic interventions (Mabire et al., 2018).

As of March 2021, based on our literature review, no systematic reviews have been conducted on dance-based interventions on depression or anxiety among persons with MCI and dementia. Only one integrative review is about dance-based interventions on anxiety among persons with dementia (Bennett et al., 2021), which included one non-RCT study and results indicate that dance might affect anxiety symptoms in patients with dementia. Given these considerations, we aimed to conduct a systematic review and meta-analysis to assess the effect of dance-based interventions on depression and anxiety for persons with MCI and dementia. We focused on two outcomes: depression as the primary outcome, and anxiety as the secondary outcome.

## Methods

### Criteria for Including and Excluding Studies for This Review

Inclusion and exclusion criteria regarding study participants, intervention, control, outcome, and study design can be found in Table 1.

**Table 1:** Criteria for including and excluding studies.

**Domain**	**Criteria for including studies**	**Criteria for excluding studies**
Population	•MCI •Dementia •Participants' formal diagnoses on types and severity of MCI and dementia were based on corresponding scales, including the Diagnostic and Statistical Manual of Mental Disorders, Fifth Edition (DSM-5) (American Psychiatric Association, 2013), International Statistical Classification of Diseases and Related Health Problems, 10th Revision (ICD-10) (World Health Organization, 1992), or other comparable diagnostic criteria, and were formally diagnosed as having any type and severity of MCI, dementia. •People of all ages •Diverse settings (e.g., the community, hospitals, nursing homes.)	•If a mixed sample of participants (e.g., dementia patients and their caregivers) was found, the data of persons with MCI and dementia were not reported separately, or the data could not be retrieved by contacting the article authors.
Intervention	•Any style of dance (e.g., tango, waltz, ballroom, polka, jazz, foxtrot, cha-cha, rumba, samba, bolero, and salsa.) •Any type of movement-with-music activities (Dance-based interventions focused on dynamic balances of the physical movements with music's rhyme and rhythm (e.g., yoga and meditation focus on the posture of the static body, thus they do not belong to dance-based interventions). The dance-based interventions included: a) 1) Non-improvised and fixed form, or structured or pre-choreographed physical movements with music (Lapum and Bar, 2016); b) or 2) improvisation of body movements following the rhythm of music; c) or 3) authentic body movements expressing mood or psychology state or subconsciously in the background of music, such as dance movement therapy (DMT). Dance-based interventions can be implemented individually, in pairs, or in groups (Ucznik and Loesche, 2017; Leach and Stevens, 2019).	•Animal trials (e.g., physical activity training of animals) •Pharmacological interventions (e.g., treatment with antidepressants).
Control group	•No intervention •Usual care (e.g., regular medication and routine care, chatted casually) •Waiting list group	•If there were more than one control group, such as music, physical, cognitive-behavioral, or integrative therapies, meta-analysis only utilized the control group of no intervention, or usual care
Outcome	•The primary outcome: Depression •The secondary outcome: anxiety •Depression and anxiety could be measured by any instruments, such as the Geriatric Depression Scale (GSD), Beck Depression Inventory (BDI), the Rating Anxiety in Dementia (RAID), Hospital Anxiety, and Depression Scale (HADS).	•The values of mean and standard deviation (SD) were not reported in the description of outcome •Mean and SD could not be obtained by contacting the authors •Mean and SD could not be calculated by review manager software or calculator provided by Cochrane (Calculator: https://training.cochrane.org/online-learning/core-software-cochrane-reviews/revman/revman-5-download; https://training.cochrane.org/resource/revman-calculator).
Study design	•Randomized controlled trials (RCTs) (including cluster-RCTs) a) Published or unpublished RCTs in Chinese or English language. b) No limitation for the year of publication.	

### Search Methods for Identification of Studies

#### Electronic Searches

Seven electronic databases (Cochrane, PsycINFO, Web of Science, PubMed, EBSCO, CNKI, WanFang) were searched from 1970 to March 2021. Grey literature was also searched and reviewed in Google Scholar, ProQuest Dissertations & Theses Database (PQDT), and Duxiu. Authors of relevant conference abstracts were reached out for possible information sharing. The search strategy was: ((MCI OR dementia OR Alzheimer's OR “cognitive impai^*^” OR “cognitive loss” OR “cognitive decline” OR (“MCI”[Mesh])) OR (“Dementia”[Mesh])) OR (“Alzheimer's Disease”[Mesh])) AND ((dance^*^ OR “authentic movement” OR “movement therap^*^” OR “movement psychot^*^” OR “body psychot^*^” OR tango OR waltz OR ballroom OR polka OR jazz OR foxtrot OR chacha OR rumba OR samba OR bolero OR salsa) OR (“Dance Therapy”[Mesh]) AND (RCT OR random^*^ OR “controlled clinical trial” OR placebo OR “drug therapy” OR trial or groups).

### Data Collection and Analysis

#### Studies Screening

The searched literature was imported into Zotero software (version 5.0.96.2) for screening, and the included and excluded literature was documented in Zotero. Six reviewers (YW, DS, YT, JW, HC, ZD) were divided into two groups, with each group member responsible for half of the articles according to inclusion and exclusion criteria. Each member independently removed duplicates, reviewed studies' titles, and abstracts, and then screened the full text. If there were disagreements and uncertainties, they were discussed at weekly group meetings with all reviewers.

#### Assessment of Risk of Bias in Included Studies

We used the Cochrane “Risk of Bias” tool (Higgins et al., 2011) to identify any risk of bias with a judgment of low risk, high risk, or unclear risk of bias for each trial of the following areas:

1. selection bias:
  a. random sequence generation
  b. allocation concealment
2. blinding of participants and personnel;
3. blinding of outcome assessment;
4. incomplete outcome data;
5. selective reporting;

Six reviewers (YW, DS, YT, JW, HC, ZD) were divided into two groups, with each group member conducted the ROB assessment for half of the articles. Each member independently conducted the ROB assessment. Disagreements and uncertainties were discussed at weekly group meetings with all reviewers.

#### Data Extraction

The data extraction was conducted manually. Six reviewers, again in two groups, independently extracted data using a pre-designed form. Each member extracted data from half of the included studies. The following data were extracted:

1. Basic study information: authors, reference, country/region;
2. Participant characteristics: illness/condition, total number, and number in each group, age, gender, race/ethnicity;
3. Intervention characteristics: intervention content, individual or group format, in-person or virtual, setting, length (e.g., number of weeks), number of sessions, duration per session, control;
4. Intervention assessment information: time point (e.g., pretest, posttest, follow-up), measures, outcomes with screenshots (including the mean, standard deviation, and the number of participants in each group at each time point), outcome raters (e.g., patients, caregivers, staff);
5. Sources of funding.

After comparing results within a group, any uncertainties that could not be solved were discussed in weekly meetings with all reviewers.

#### Data Analysis

RevMan 5.4 was used for meta-analysis. Outcomes measured in at least two studies were included in the meta-analysis. Heterogeneity was assessed and interpreted using an *I*^2^ statistic according to Cochrane guidance: 0–40% means not important; 30–60% represents moderate heterogeneity, 50–90% represents substantial heterogeneity; and 75–100% is considerable heterogeneity (Higgins et al., 2020). Given the possible clinical heterogeneity, a random-effects model was used. If one study used more than one instrument to measure the same outcome variable, the team employed the more commonly used instrument for the analysis. Subgroup analyses were conducted with the following characteristics if applicable: outcome instrument, disease/conditions, country/region, setting, frequency, session, post-intervention, follow-up, raters. Cohen's d was calculated for subgroup analyses to compare the effect size. A funnel plot was used to assess publication biases when the number of studies used for meta-analysis was more than ten, according to Cochrane guidelines.

### Rating the Evidence Quality

We used the tool of GRADEpro GDT (Schünemann et al., 2013) to rate the evidence quality of meta-analysis results in five aspects: risk of bias, inconsistency, imprecision, indirectness, and publication bias, according to Cochrane guidelines, and then we chose whether to downgrade or not. The final evidence quality was judged as high, medium, low, or very low. Different opinions on the rating were discussed and finally decided at the weekly meetings.

## Results

### Search Results

**Figure 1:**
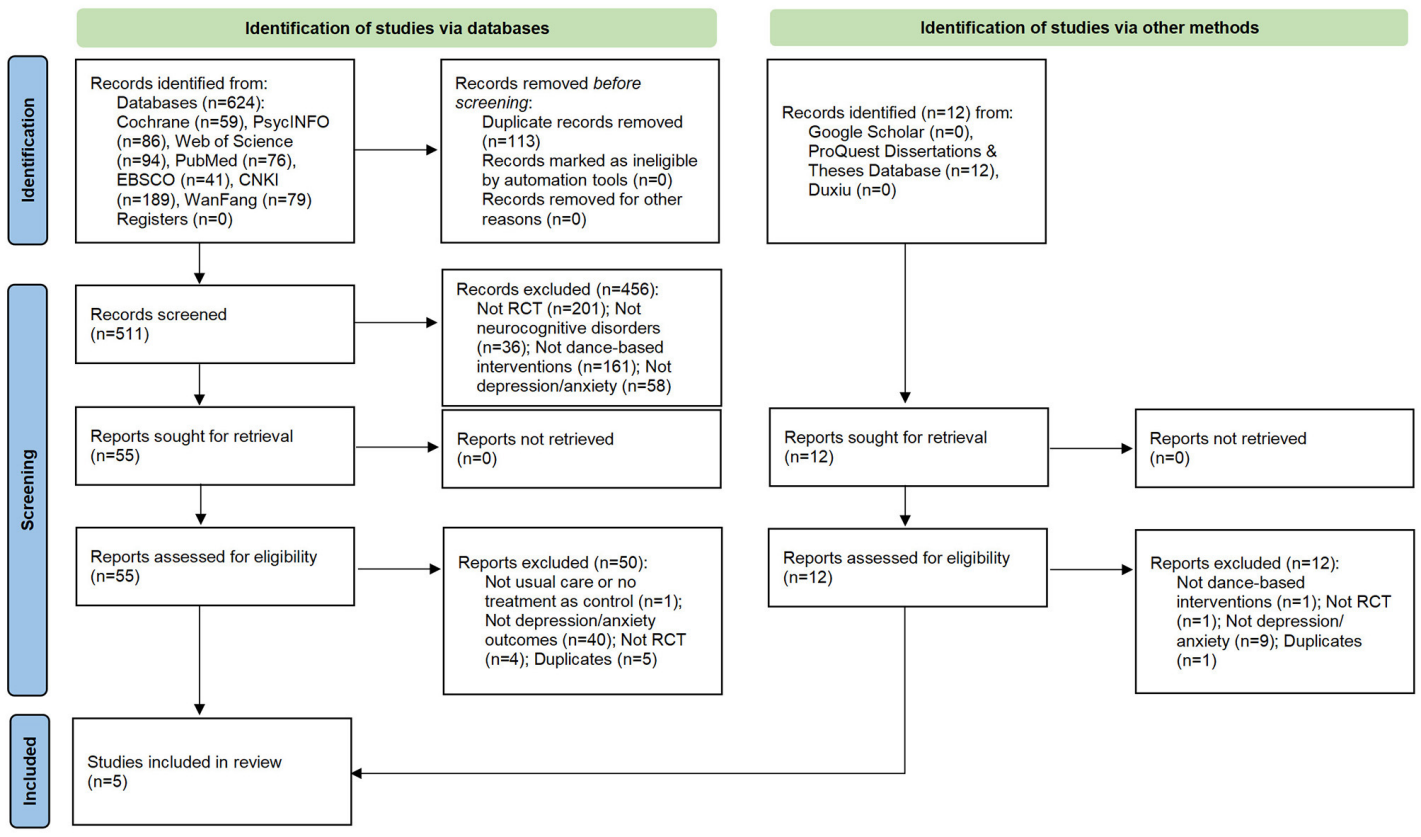
Flowchart of the study selection process.

Figure 1 shows the PRISMA flowchart of the study review and selection process. A total of 563 studies were identified from electronic searches. These studies were de-duplicated and screened at the title and abstract level with the pre-stated inclusion and exclusion criteria. Fifty-five full-text articles were then screened, among which 50 were excluded based on the same criteria. As a result, five studies representing 579 participants were included in the systematic review and meta-analysis.

### Study Characteristics

Table 1 presents the characteristics of included studies. Among five studies, three were conducted in China (one in mainland China and two in Hong Kong), one in Greece, and one in the United States. All five studies were RCTs.

Regarding participants' diseases/conditions, participants in most studies (*n* = 3) were persons with MCI (Aguiñaga, 2016; Lazarou et al., 2017; Zhu et al., 2018), one was persons with moderate dementia (Cheung et al., 2018), and one was mild dementia (Ho et al., 2018). The total number of participants ranged from 21 to 204, with three studies having more than 100 participants. Participants from two studies were community residents (Aguiñaga, 2016; Lazarou et al., 2017); one was recruited from residential care facilities (Cheung et al., 2018); one was from a local hospital and older adult community centers (Ho et al., 2018); and one was from a dementia clinic as well as through radio and newspaper recruitment ads (Zhu et al., 2018).

In terms of the intervention details, the included studies were all group-based interventions. Intervention length ranged from 6 weeks to 12 months. The frequency of each intervention included 20 min twice a week (*n* = 1), 35 min three times a week (*n* = 1), and 60 min twice a week (*n* = 3). All interventions were conducted with persons older than 50 years old. The intervention sessions ranged from 12 to 80 sessions, including 12 sessions (Cheung et al., 2018), 24 sessions (Ho et al., 2018), 32 sessions (Aguiñaga, 2016), 36 sessions (Zhu et al., 2018), and 80 sessions (Lazarou et al., 2017).

Regarding dance-based intervention approaches, there are two main approaches: one is dance movements therapy (DMT) with improvisation elements led by a registered dance-movement therapist. This approach includes four main elements: simple group dance, movement games, improvisational dance movement, and movement interactions among group members (Ho et al., 2018). The other approach is non-improvised, fixed form and structured movements with music, including music-with-movement, international ballroom dancing, BAILAMOS© program, and aerobic dance. Specifically, music-with-movement was delivered by a well-trained interventionist using batting balloons, waving ribbons, foot-tapping, playing musical instruments, and mimicking movements (Cheung et al., 2018). International ballroom dancing was led by an experienced dance instructor with a 5-min warm-up, 45 min of new material (figures/dances), and a 10-min cool-down. The instructor paid attention to a dance of participants' preference, taught them to recognize music, dance, and rhythm, and finally remember step combinations, leading hints, and following alertness (Lazarou et al., 2017). BAILAMOS© program was an innovative, culturally appropriate dance program developed by Dr. Marquez and an accomplished Latin dance instructor. When used in the study, it was revised and practiced by a professional dance instructor (Aguiñaga, 2016). Aerobic dance was taught by a dance instructor with a 5-min warm-up, a 25-min dance with the target heart rate, and a 5-min cool-down. Seven sub-sessions were performed consecutively: knee bending, heel up, boxing, shoulder movement, kicking, square-stepping, and sculling exercises (Zhu et al., 2018).

Control groups included usual care in one study (Zhu et al., 2018), and participants' usual lifestyle/usual activities in two studies (Aguiñaga, 2016; Lazarou et al., 2017). In the remaining two studies, two control groups were used. Specifically, in Ho et al. (2018), one group was waitlist control, and the other was a moderate exercise program. In Cheung et al. (2018), one group was social activity group (chatted casually), and the other was a music listening group.

Regarding funding sources, four studies reported the source of funding (Aguiñaga, 2016; Lazarou et al., 2017; Ho et al., 2018; Zhu et al., 2018), which were all educational and research grants. The remaining one study reported receiving no funding (Cheung et al., 2018).

Regarding measurement instruments, depression was measured in all five of the included studies, with measurement tools including the Geriatric Depression Scale (GDS), GDS-15, and four dichotomous items of the GDS. Anxiety was measured in one included study which used the Rating Anxiety in Dementia (RAID). As for measurement time points, all five studies had pre-and post-intervention assessments. Four studies had follow-up (f/u) assessments, and the f/u assessments were conducted at various time points (e.g., 3 months, 4 months, 6 months, 8 months, and 12 months) (Table 2).

**Table 2:** Characteristics of included studies.

**Study reference**	**Participants**	**Study design**	**Intervention Group (IG)**	**Control Group (CG)**	**Outcome measures**	**Data collection time and raters**	**Results**	**Sources of funding**
Cheung et al. (2018) The effects of the music-with-movement intervention on the cognitive functions of people with moderate dementia: A randomized controlled trial	*N* = 165 Age ≥ 65 y/o Individuals with moderate dementia from 12 residential care facilities in Hong Kong, China	RCT	*N* = 58 each group: 4–6 persons 6-week music-with-movement (MM) intervention happening twice a week, with 20 min in each session. The first author served as the interventionist and was well-trained in delivering the intervention, including batting balloons, waving ribbons, foot-tapping, playing musical instruments (e.g., handbells, drums, triangles, etc.), and mimicking movements 12 session	*n* = 54, music listening group (listened to their preferred music) *n* =53, social activity group (SA, chatted casually)	Depression: GDS Anxiety: RAID	T_0_ (baseline) T_1_ (6 weeks) T_2_ (6 weeks post-intervention, 12 week) Rated by research staff	T0-T1: GDS (MM: *p* = 0.002 SA: *p* = 0.194) RAID (MM: *p* <0.001 SA: *p* = 0.128) TI-T2: GDS (MM: *p* = 1.000 SA: *p* = 0.078) RAID (MM: *p* = 0.873 SA: *p* = 0.626) T0-T2: GDS (MM: *p* = 0.052 SA: *p* = 0.922) RAID (MM: *p* < 0.001 SA: *p* = 0.001)	No funding
Lazarou et al. (2017) International ballroom dancing against neurodegeneration: A randomized controlled trial in Greece community-dwelling elders with mild cognitive impairment	*N* = 129 Age: 55–75 y/o, mean age 66.8 ± 10.1 Community residents with MCI in Greece	RCT	*N* = 89: received allocated intervention (*n* = 74); did not receive allocated intervention (*n* = 15); lost to follow-up (*n* = 5); discontinued intervention (*n* = 3); analyzed (*n* = 66) International Ballroom Dancing; Group practice; Twice a week for 60 min for 10 months, 40 weeks in a total of 80 sessions per person. An experienced dance instructor supervised the dance class. Each 60-min dance class included a 5-min warm-up revising previous dance sessions, 45 min of new material (figures/dances), and a 10-min cool-down period with a dance of participants' preference; recognize music, dance, and rhythm, to stay in the rhythm in basic simple steps, and finally remember step combinations, leading hints and following alertness.	*N* = 65: lost to follow-up (*n* = 2), analyzed (*n* = 63), no intervention, continued participants' usual lifestyle	Depression: GDS, BDI	Pre, post, and after the end of the program-−10 months later Rated by researchers.	GDS: Post: *p* = 0.022 The independent sample *t*-test at the follow-up: GDS *p* = 0.007; DBI *p* = 0.04	The project “Augmentation of the Support of Patients suffering from Alzheimer's Disease and their caregivers (ASPAD/2875)”, The European Union (European Social Fund) and the Ministry of Education, Lifelong Learning and Religious Affairs in the context of the National Strategic Reference Framework (NSRF, 2007-2013).
Aguiñaga (2016) Latinos unique scenario, addressing cognitive impairment via dance	*N* = 21 Age > 60 y/o, mean age 75.4 ± 6.3 Community-dwelling older Latinos with MCI (score 18–26) in Chicago, U.S.	RCT	*N* = 10, completers *n* = 6 16-week (four months), 1 h twice a week 32 sessions. BAILAMOS© program is an innovative, culturally appropriate dance program and has been developed by Dr. Marquez and an accomplished Latin dance instructor, and revised and practice by a professional dance instructor.	*N* = 11, the wait-list control group were asked to maintain their usual activities during weeks 1 through 16	Depression: GDS-15	Testing occurred at baseline, 2 months, 4 months, 6 months, and 8 months Rated by the research staff member	The GDS-15 showed an interaction effect, [*F*_(1.61, 30.67)_ = 5.76, *p* = 0.01] in the direction contrary to hypothesize, that is the intervention will yield small-medium effect sizes reflecting improvements om depression	The University of Illinois at Chicago Department of Kinesiology and Nutrition, and the Rush Alzheimer's Disease Center
Ho et al. (2018) Psychophysiological effects of dance movement therapy and physical exercise on older adults with mild dementia: A randomized controlled trial	*N* = 204 Age ≥ 65 y/o Individuals with a clinical diagnosis of mild dementia from psychogeriatric outpatient departments of a local hospital and older adults community centers in Hong Kong, China	RCT	*N* = 69 DMT, group, 12 weeks, with 1-h sessions held twice a week, led by registered dance-movement therapist or one in training; 24 session. Four main elements: simple group dance, movement games, improvisational dance movement, and movement interactions among group members.	Exercise group: *n* = 67, mild to moderate exercise program, 2 h per week (12 weeks) mild to moderate exercise program (a warm-up, stretching, and joint movements, exercising with towels and a cooldown) Control group: *n* = 68, the waitlist control group received regular medication and routine care	Depression: four dichotomous items of GDS	T_1_ (baseline) T_2_ (3 months) T_3_ (6 months) T_4_ (1 year) Research coordinators+ Participants completed self-rated scales	From Time 1 to Time 2, the DMT group showed significantly lower scores in depression (*B* = −0.51, SE = 0.19, *p* < 0.01, *d* = 0.33) than the control group. From Time 2 to Time 4, however, the DMT group showed considerable rebounds in depression, At Time 4, the DMT and control group no longer differed significantly in these three variables (*d* = 0.10–0.21, *p* = 0.08–0.42).	The General Research Fund, Hong Kong Research Grants Council (GRF/HKU17402714).
Zhu et al. (2018) Effects of a specially designed aerobic dance routine on mild cognitive impairment	*N* = 60 Age ≥ 50 y/o and ≤ 85 y/o MCI patients recruited through the dementia clinic at the First Affiliated Hospital of Nanjing Medical University or through radio and newspaper recruitment ads inNanjing, China	RCT	*N* = 29; Aerobic dance taught by a dance instructor including a 5-min warm-up, a 25-min dance with the target heart rate, and a 5-min cool-down; seven sub-sessions performed consecutively: knee bending, heel up, boxing, shoulder movement, kicking, square-stepping, and sculling exercise; 35-min dance session, three times a week for 3 months; 36 session. 3 months later continue practicing in their own home	*N* = 31, usual care only	Depression: GDS-15	T_0_ (baseline) T_1_ (3 months) T_2_ (6 months) Rated by physicians and technician	Patients in the control group had a significant improvement at 3 months in depression symptoms (mean GDS-15 score change −3.4, 95% CI −5.2, −1.5; p, 0.01) but no significant at other time points.	The Science and Technology Department of Jiangsu Province (Project Number: 2013-DB13)

*BDI, Beck Depression Inventory; GDS, Geriatric Depression Scale; RAID, The Rating Anxiety in Dementia; HADS, Hospital Anxiety and Depression Scale*.

### Risk of Bias

**Figure 2:**
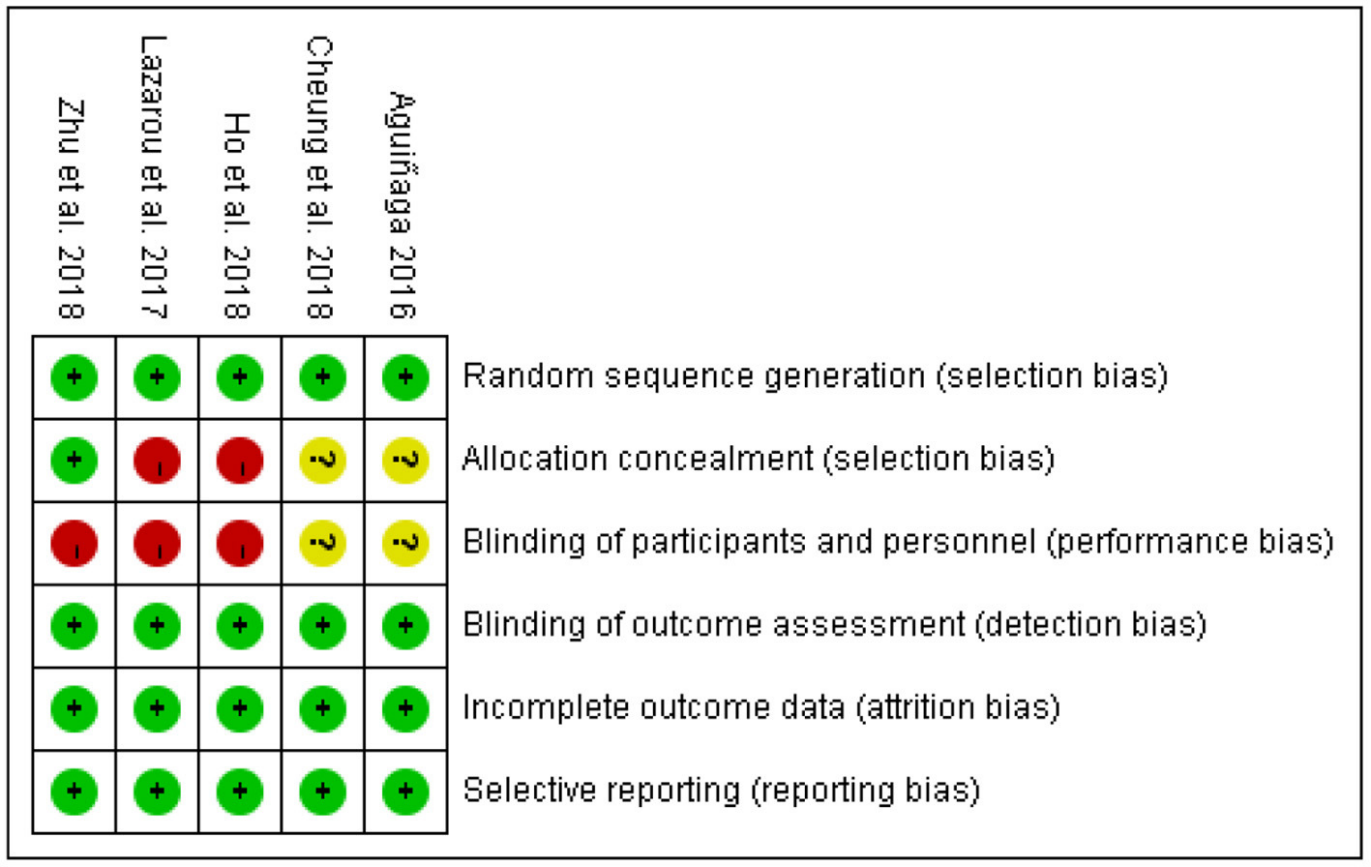
Risk of bias summary. *Green represents low risk; Red represents high risk; Yellow represents unclear.

Risks of bias were judged based on the Cochrane guidance, as shown in Figure 2. Regarding random sequence generation, five studies were judged to be at low risk, as they used computer-generated random numbers (Aguiñaga, 2016; Ho et al., 2018; Zhu et al., 2018), or block randomization method (Cheung et al., 2018), or a technician performed randomization using an algorithm (Lazarou et al., 2017).

Regarding allocation concealment, only one of five studies was judged to be at low risk, which used sealed envelopes (Zhu et al., 2018). Two studies were judged as high risk which reported that no concealment was used in the allocation process (Aguiñaga, 2016; Ho et al., 2018). The remaining two studies did not report information on allocation concealment and were judged to be unclear.

Regarding blinding participants and interventionists, three studies (Lazarou et al., 2017; Ho et al., 2018; Zhu et al., 2018) were rated as high risk, and two studies (Aguiñaga, 2016; Cheung et al., 2018) as unclear. Meanwhile, all five studies were judged as low risk for blinding outcome assessment through using personnel not included in the intervention process.

All studies were judged to be at low risk for incomplete outcome data because the dropout rate was low (<30%) during the intervention. All explained the numbers and reasons for dropout and the data analysis methods of deal with missing values. Lastly, all studies were judged as low risk for selective reporting.

### Meta-Analysis Results for Depression

**Figure 3:**
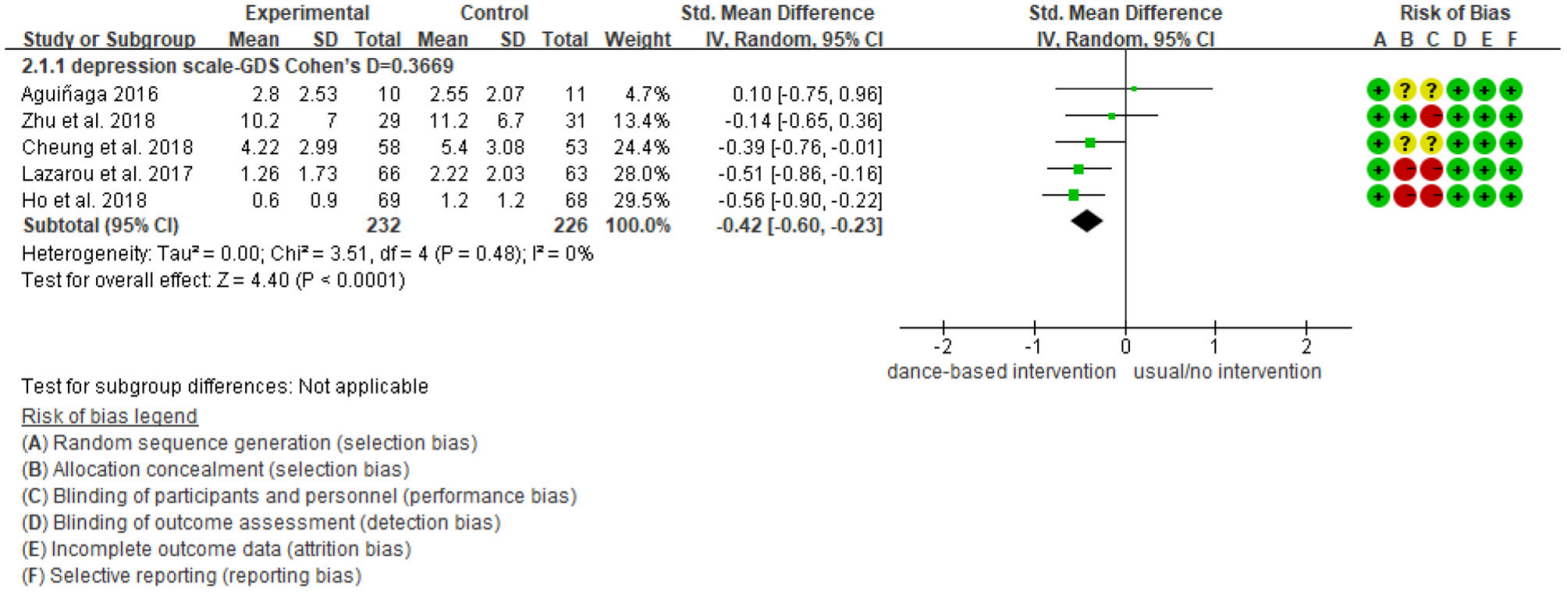
Effect size of the intervention group vs. the control group on depression rating scores.

As shown in Figure 3, five studies reported data on depression and were pooled for a meta-analysis using the random-effects model. Results showed no heterogeneity (I^2^ = 0%), meaning that the variation in study outcomes did not influence the interpretation of results. There were significant standardized mean differences in favor of dance-based interventions compared with controls for depression [SMD = −0.42, 95% CI (−0.60, −0.23), *p* = <0.05] at the time point of post-intervention (The intervention length in the five included studies were 3 months, 4 months, 10 months, 6 weeks, respectively). The result indicated that dance-based interventions were significantly beneficial to persons with MCI and dementia compared with the control group, although the effect size was small (Cohen's *d* = 0.3669).

**Figure 4:**
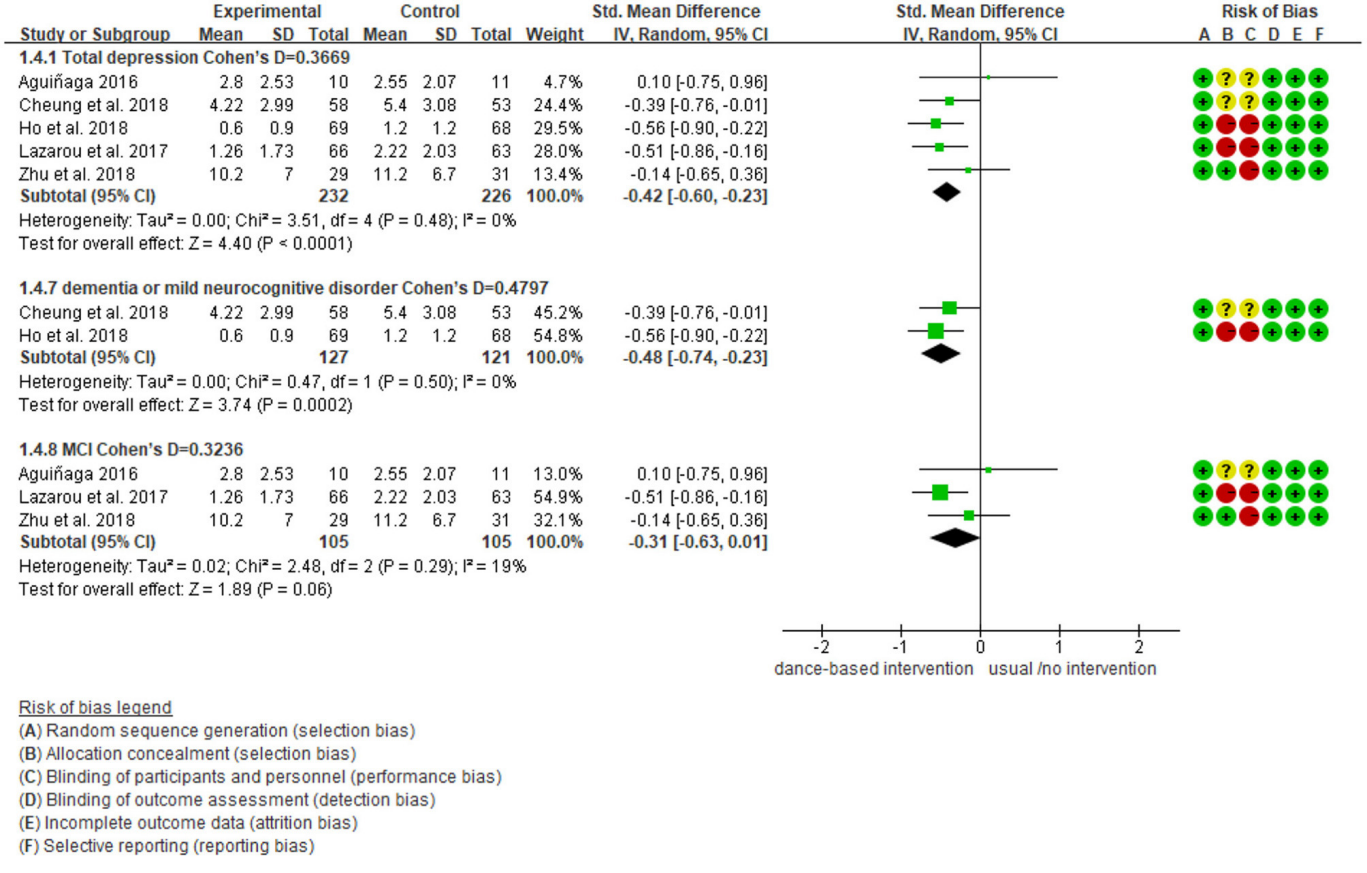
Subgroup analyses of the intervention group vs. the control group on depression rating scores: Disease.

**Figure 5:**
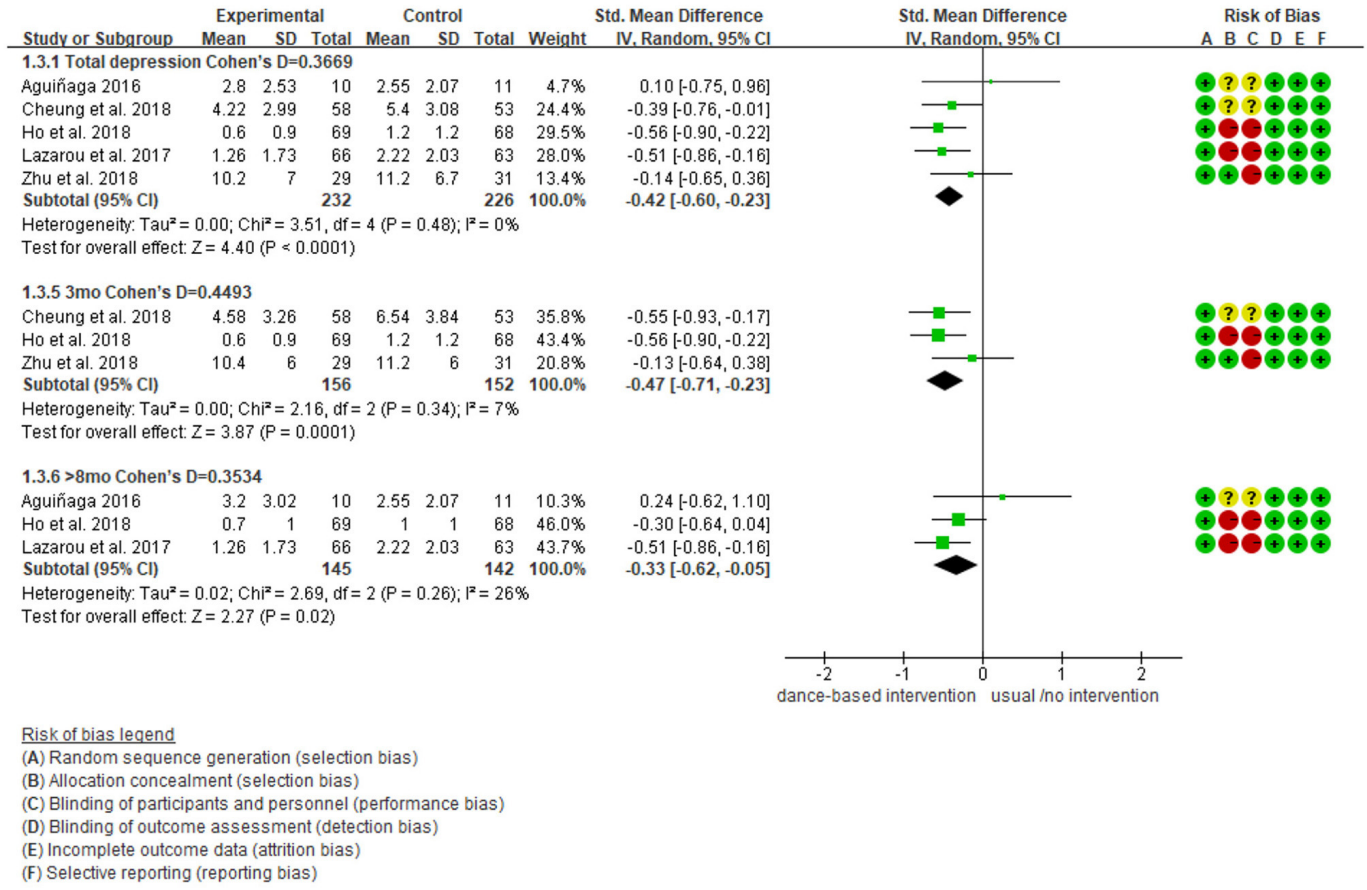
Subgroup analyses of the intervention group vs. the control group on depression rating scores: Intervention length.

**Figure 6:**
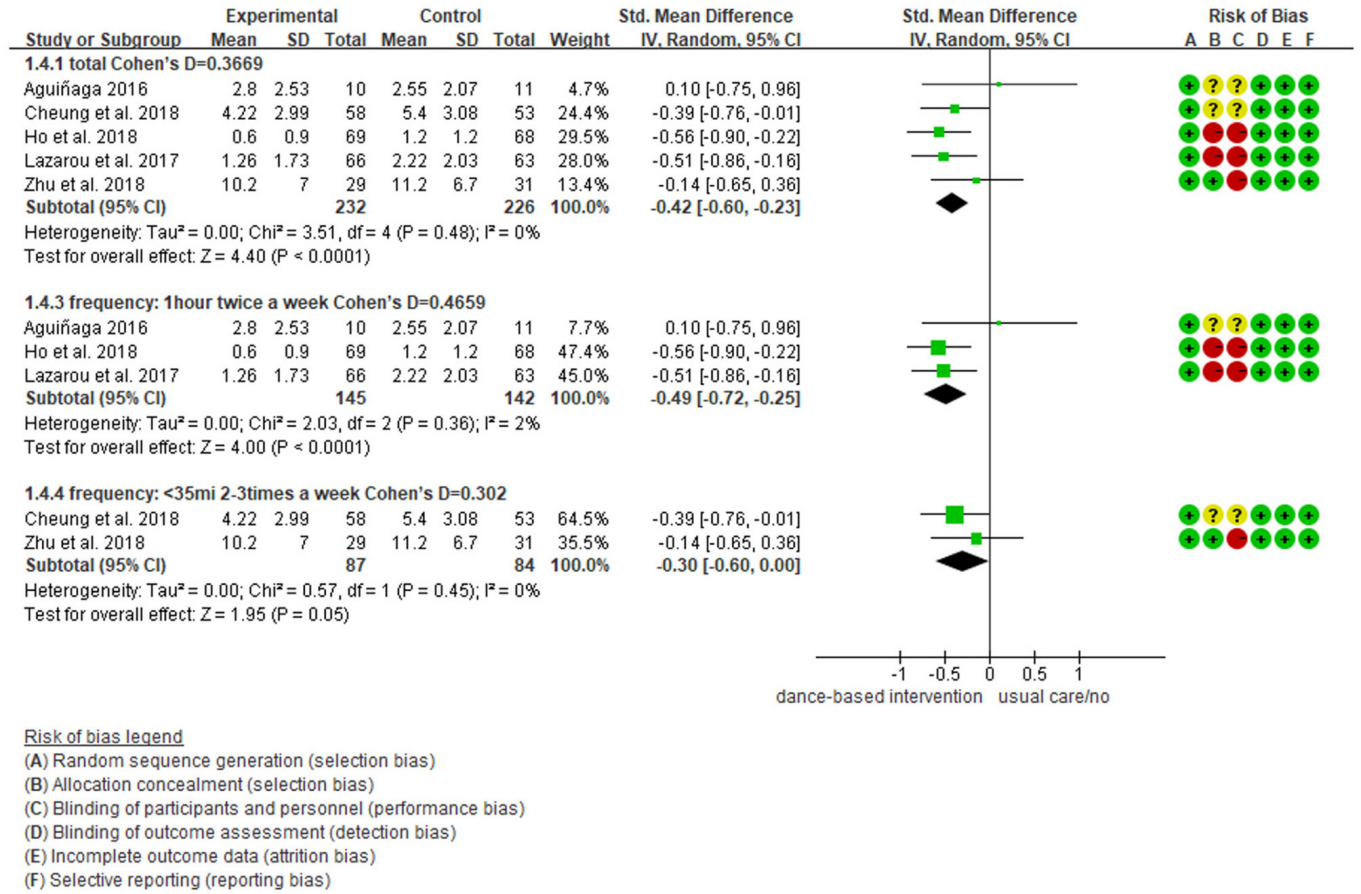
Subgroup analyses of the intervention group vs. the control group on depression rating scores: Intervention frequency.

**Figure 7:**
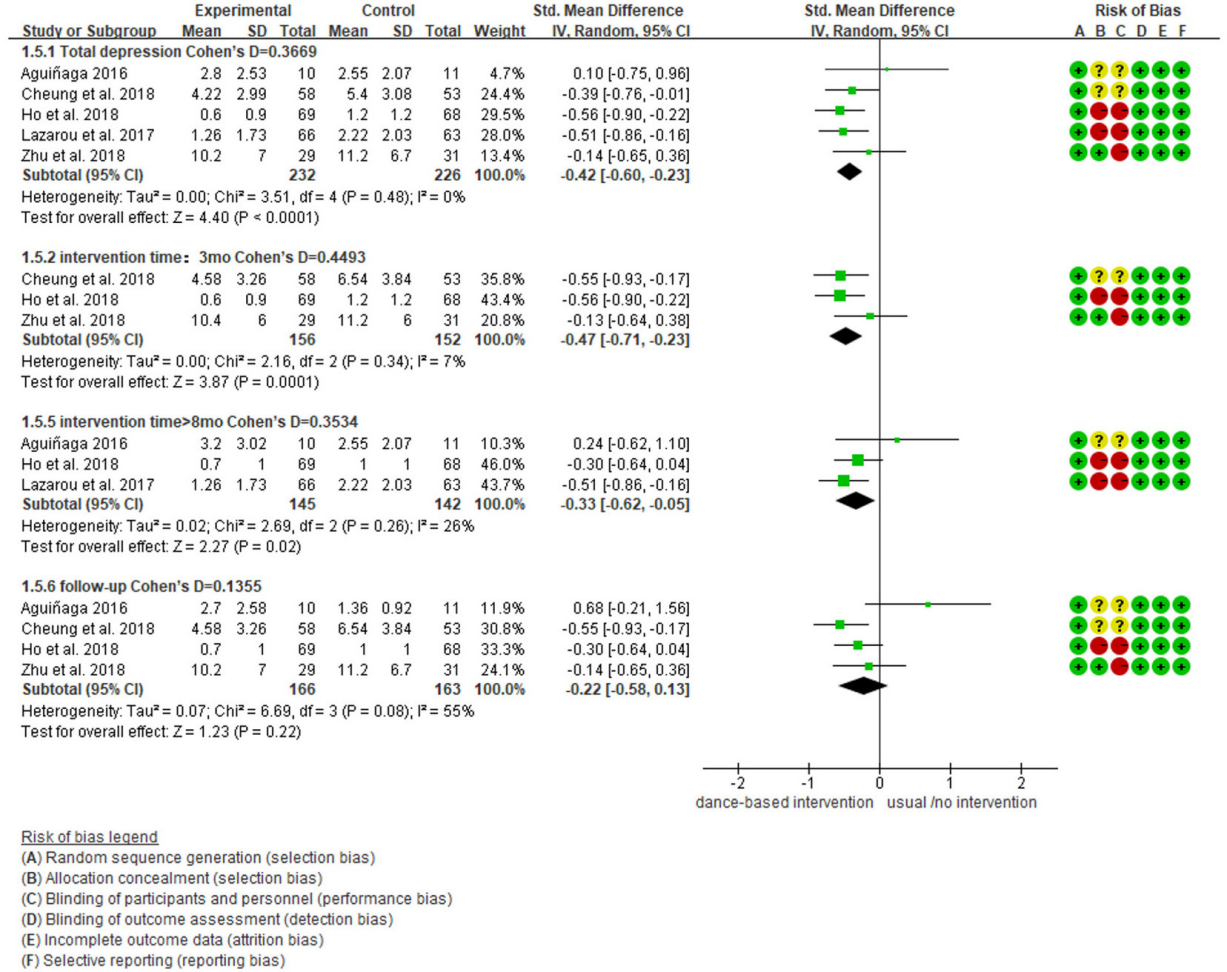
Subgroup analyses of the intervention group vs. the control group on depression rating scores: Time points of data collection.

**Figure 8:**
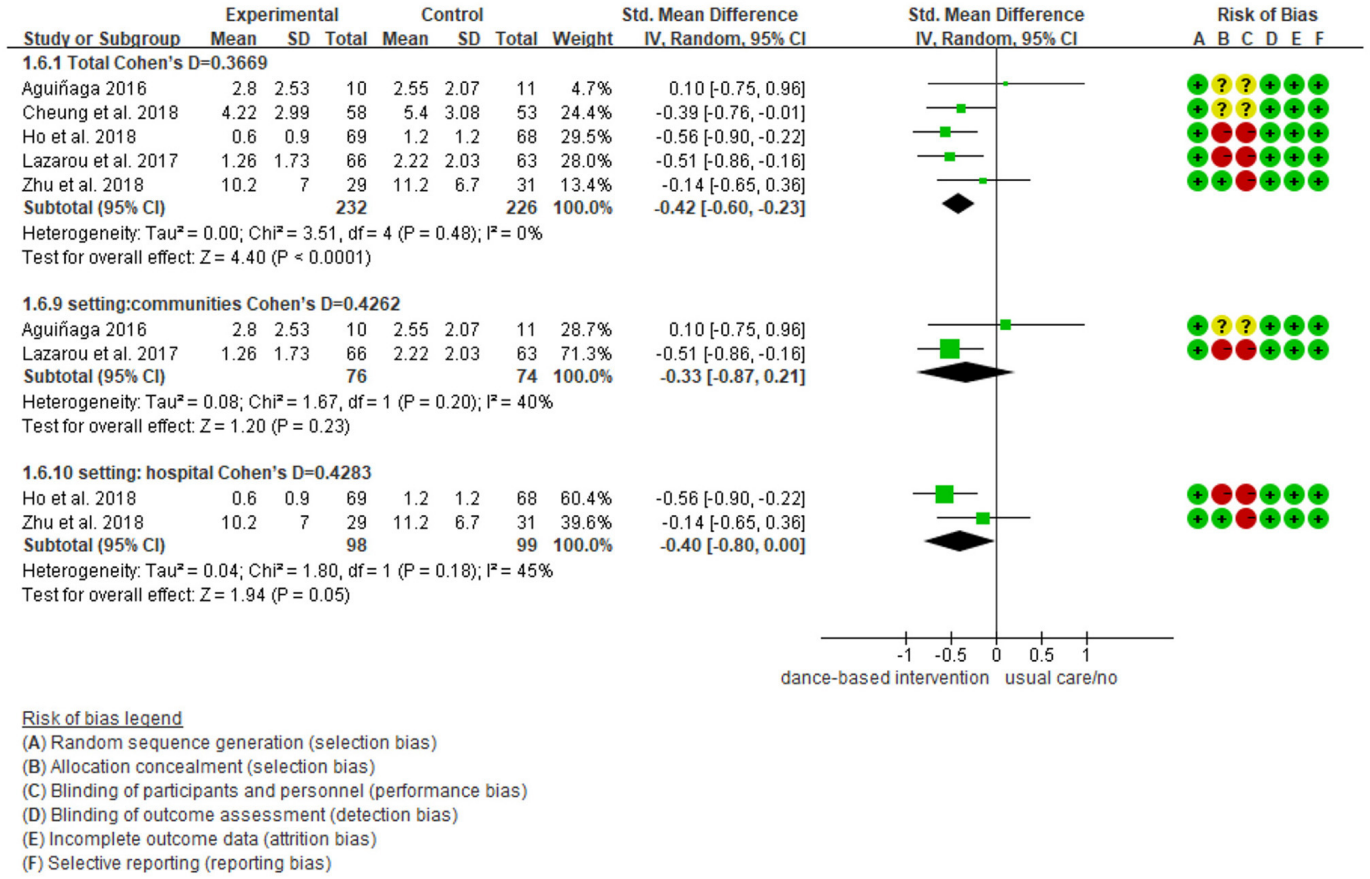
Subgroup analyses of the intervention group vs. the control group on depression rating scores: Intervention setting.

**Figure 9:**
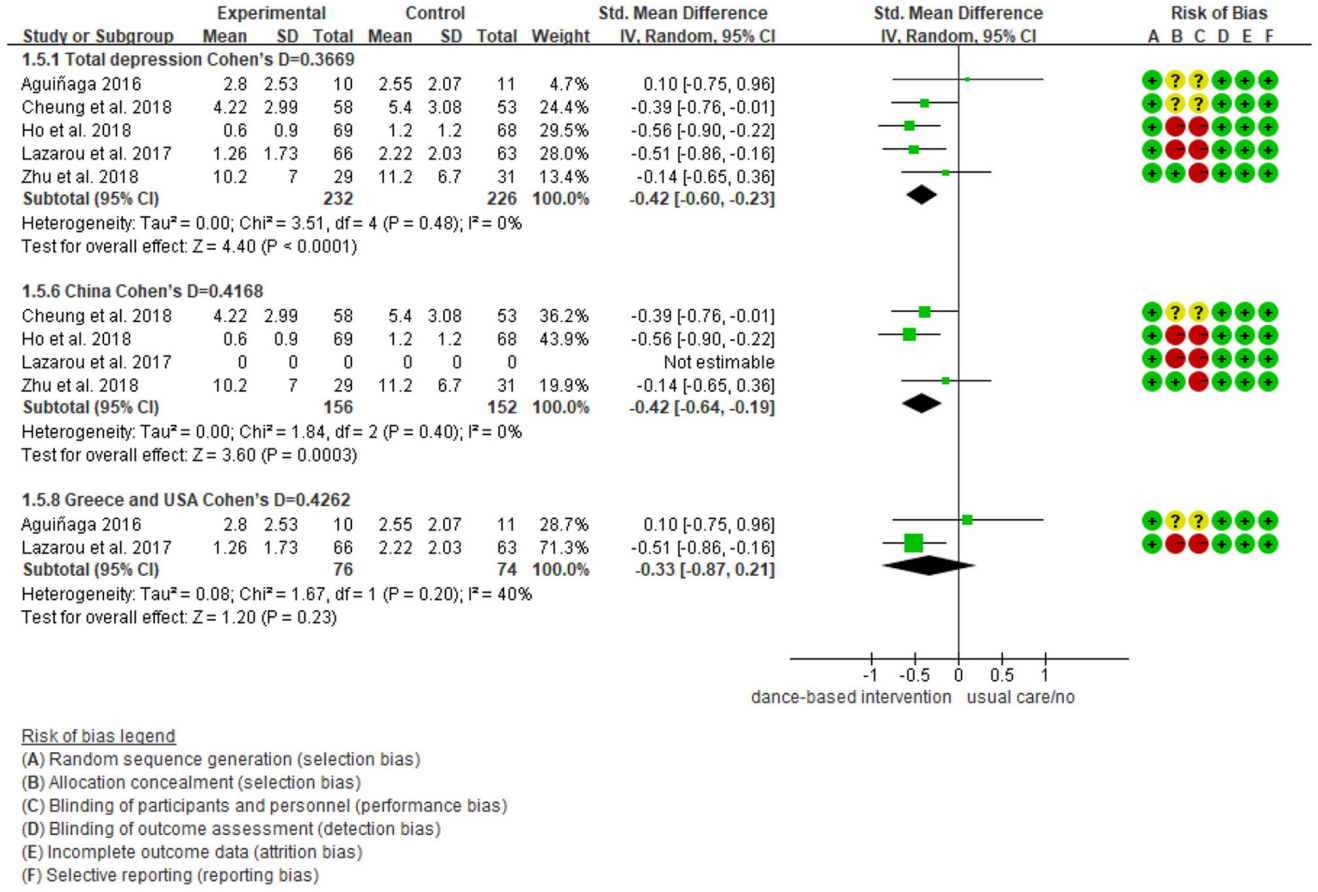
Subgroup analyses of the intervention group vs. the control group on depression rating scores: Intervention countries.

We further performed subgroup analyses with results showing in Figures 4–9. Overall, results showed heterogeneity ranged from 0 to 55%, showing small or moderate heterogeneity according to Cochrane guidance. The subgroup analysis for the assessment points of follow-ups showed moderate heterogeneity (*I*^2^ = 55%).

Subgroup analyses showed Cohen's d ranged from 0.1355 to 0.4797, indicating that the effect of dance-based interventions tended to be small or medium in different conditions. Specifically, in terms of disease (Figure 4), studies with MCI patients had a smaller effect size than those with dementia patients (0.3236 vs. 0.4797). In terms of intervention length (Figure 5), studies with 8 months had a smaller effect size than that of 3 months (0.3534 vs. 0.4493).

In terms of intervention frequency (Figure 6), studies with 35 min 2–3 times a week had a smaller effect size than that of 1 h twice a week (0.3020 vs. 0.4659). In terms of time points of data collection (Figure 7), studies assessed at post-intervention had a larger effect size than those assessed at follow-ups (0.3669/0.4493/0.3534 vs. 0.1355), which showed that dance-based interventions had diminishing effects. In terms of intervention settings (Figure 8), studies in hospitals and communities showed similar effect sizes (0.4262 vs. 0.4283). In terms of intervention countries (Figure 9), studies in China showed a similar effect size to non-Asia studies (combining Greece and the United States) (0.4168 vs. 0.4262).

### Analysis of Publication Bias for Depression

Only five RCTs were included, so the funnel plot was not made and publication bias was undetected; however, it could not be ruled out.

### Intervention Effects on Anxiety

**Figure 10:**
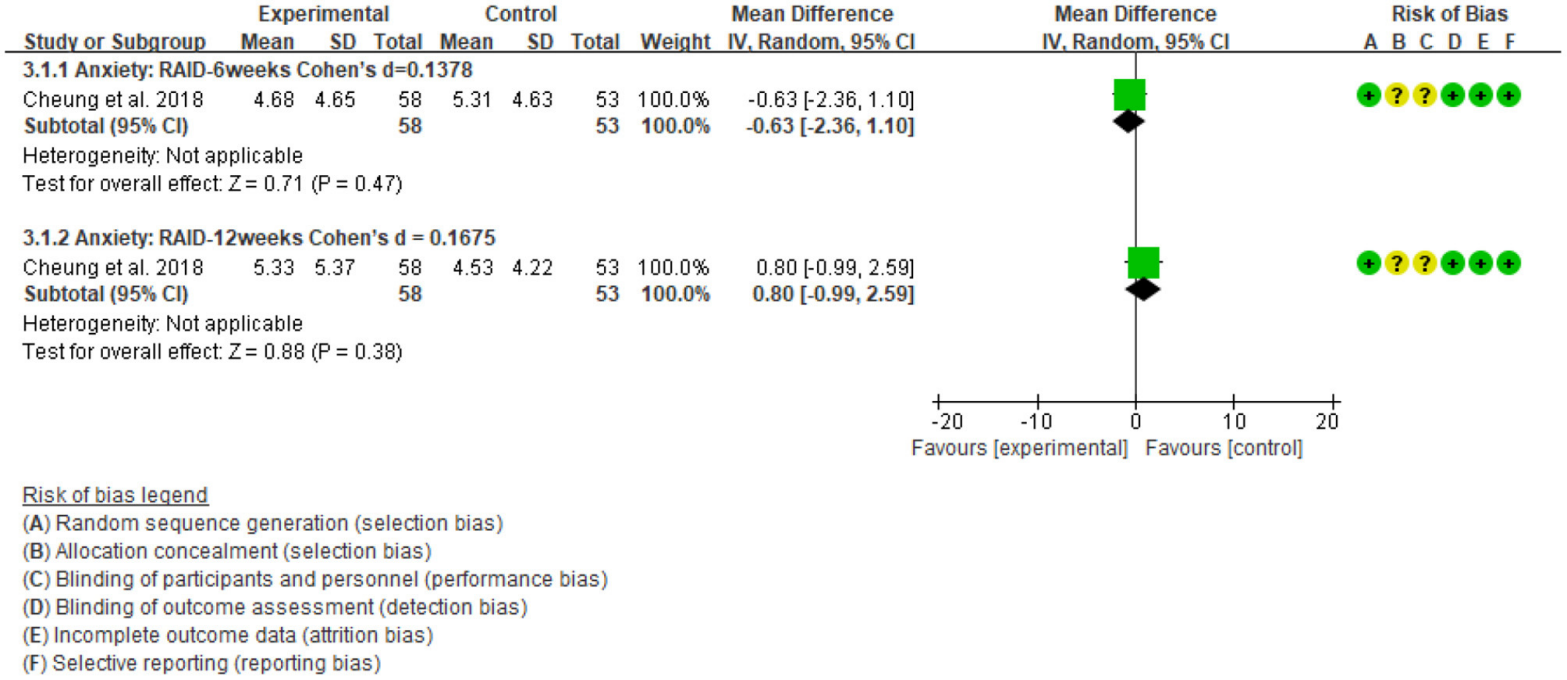
The intervention group vs. the control group on anxiety rating scores.

One included study (Cheung et al., 2018) reported data on anxiety representing 165 older adults with moderate dementia. Results showed that there were no significant standardized mean differences in favor of the dance-based intervention compared with the control for anxiety at post-intervention [MD = −0.63, 95% CI (−2.36, 1.10), *p* = 0.47], with a small effect [Cohen's *d* = 0.1378]. Also, the result was not significant at the 6-week follow up [MD =0.80, 95% CI (−0.99, 2.59), *p* = 0.38], although a small effect existed [Cohen's *d* = 0.1675] (Figure 10).

### GRADE Evidence Quality Rating

**Figure 11:**
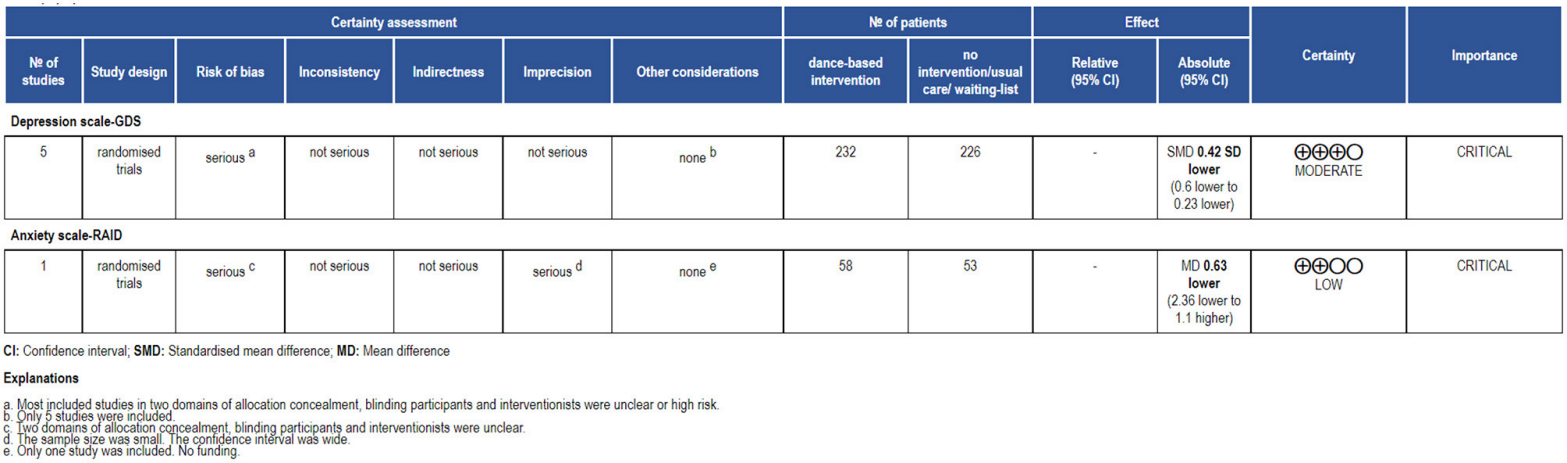
GRADE evidence quality rating of depression and anxiety.

Figure 11 shows the evidence quality of the depression and anxiety outcomes rated by GRADE. The final judgment of the evidence quality of depression outcome was moderate. This was related to the risk of bias rated as serious because most included studies were judged to be unclear or high risk in the two domains of allocation concealment, and blinding participants and personnel. Also, the evidence quality of the anxiety outcome was low. This was related to risk of bias and imprecision. The risk of bias was judged to be serious because the included study (Cheung et al., 2018) was assessed as unclear in the two domains of the allocation concealment, and blinding participants and personnel. Imprecision was judged to be serious because the study had a small sample size (*n* = 53) which did not meet the requirements of optimal information size (OIS); the confidence interval was wide [CI (−2.36, 1.10)]. These led to a degradation in the evidence quality due to precision (Guyatt et al., 2011).

## Discussion

This systematic review and meta-analysis study examined the effects of dance-based interventions on depression and anxiety among persons with MCI and dementia. A total of five studies representing 579 participants were identified. As shown in results, dance-based interventions are significantly beneficial to persons with MCI and dementia in decreasing depression compared with controls. The evidence quality of depression outcome is moderate. Also, dance-based interventions are shown to be beneficial but not significant to persons with moderate dementia in decreasing anxiety compared with controls. The evidence quality of the anxiety outcome is low.

Our review confirms that dance-based interventions are important and feasible to cope with depression for persons with MCI and dementia. Consistent with our findings, the meta-analysis on health-related psychological outcomes by Koch et al. (2019) suggests that DMT can improve the symptoms of depression; the systematic review on physical and mental illnesses from Kiepe et al. (2012) also shows that patients with depressive psychological distress can be alleviated by dance therapy. However, the review of Koch et al. (2019) shows high heterogeneity of included studies; both Koch et al. (2019) and Kiepe et al. (2012) examined diverse diseases, and depression was assessed in the general populations, not with persons with MCI and dementia. Our review includes RCTs and proves the significance of the effect on depression for persons with MCI and dementia.

In this review, we further identified practice characteristics of dance-based interventions. For example, practitioners should be sensitive to the decreasing effect of interventions. That is, the effect at the post-intervention might be more effective than that of follow-ups. This result also suggests that its current intervention levels of frequency and duration might not be sufficient to yield a long-lasting effect.

Meanwhile, the effects of dance-based interventions are better for persons with dementia than those with MCI. It could be because individuals with MCI represent the earliest symptomatic stage of dementia, and not all individuals with MCI would progress to dementia (Morris, 2005). Thus, depressive symptoms in persons with MCI are lighter than those with dementia. However, patients with MCI and concomitant depression/anxiety have more pronounced cognitive deficits and progress more often to dementia than MCI patients without depression/anxiety (Ma, 2020). Thus, the interventions for decreasing negative emotions and psychological symptoms should be carried out as early as possible. Also, we recommend the intervention frequency of an hour twice a week.

Additionally, we suggest that practitioners must consider the safety of dance-based interventions because falls are a key risk among persons with MCI and dementia who typically also have high frailty levels (Van Doorn et al., 2003; Fernando et al., 2017). Besides, our review shows that there is a similar effect of dance-based interventions in both hospital and community settings, indicating the interventions can be carried out in various settings to achieve their beneficial effects. Besides, although meta-analysis shows no statistical heterogeneity, we would like to emphasize that statistical heterogeneity could only reflect clinical and methodological heterogeneity to a certain extent. There is no statistical heterogeneity, but it does not mean that there is no clinical and methodological heterogeneity. For example, effects are likely to be different if dance-based interventions are conducted 6 weeks or 10 months, likewise 12 or 80 sessions, or the wildly differing lengths of follow-up. Given the clinical heterogeneity, we call for more studies to provide more clinical guidance for practitioners.

Regarding the anxiety outcome, our review only identified one RCT study. Results suggest that the dance-based intervention is beneficial but not statistically significant in reducing anxiety in persons with moderate dementia compared with the control. Consistent with our review, the integrative review (Bennett et al., 2021) shows the same result, but it included one non-RCT study. This means that we need more evidence to confirm the effect of dance-based interventions on anxiety among persons with MCI and dementia. Notably, anxiety is a risk factor for persons with MCI and dementia (Rosenberg and Lyketsos, 2013; Somme et al., 2013), and dance-based interventions as a type of embodied psychological intervention can address mental symptoms (Wang et al., 2018; Wu et al., 2019). However, a presentation of anxiety in the context of MCI and dementia can be different from a typical early-onset anxiety disorder, and it is not easy to identify and quantify anxiety reliably (Kwak et al., 2017). Thus, more research on effects of dance-based interventions on anxiety is needed.

In this review, we further used GRADE to rate evidence quality and identified the moderate quality evidence of depression outcome and low-quality evidence of anxiety outcome. It is noteworthy that the risk of bias is one of the crucial reasons for evidence degradation for both depression and anxiety outcomes. Only one study reported adequate allocation concealments and the risk bias was rated as low in this domain; no study was rated as low risk in blinding of participants and personnel. This can generate bias as unblinded interventionists might exaggerate the intervention effects, and the intervention might have a placebo effect on the participants.

Our study has several limitations. First, this review only included five studies, and publication bias may exist. Second, we only included English and Chinese literature and thus excluded potential useful information written in other languages. Third, this study might be limited by the selected databases. Although this review included the most widely used English and Chinese databases, it remains possible that some works, particularly unpublished studies conducted in other countries, were not located and examined. Also, our review aimed to assess the effect of dance-based intervention in alleviating depression and anxiety compared with non-intervention/usual care/waitlist control group, so the review can not prove whether dance-based interventions are more or less effective than other therapies. Additionally, we recognize the limitation that GDS measures depressive symptomatology, but it is not clear whether the patients were clinically depressed. More attention should be paid to the measurement instrument in the future. Finally, we did not get a chance to register our protocol on PROSPERO which may lead to bias.

Future research implications are as follows. First, more well-designed RCT studies are warranted to evaluate the effects of dance-based interventions among persons with MCI and dementia. The outcome should include not only depression and anxiety, but also other psychosocial outcomes that are crucial to one's holistic well-being. We also call for more rigorous design, especially high-quality designs that minimize biases in domains of allocation concealment and blinding of participants and personnel. In addition, a variety of studies with a larger sample size is necessary to clarify the efficacy. Meanwhile, more rigorous trials that investigate the effect of dance-based interventions during a longer time period should be undertaken, in order to monitor the ongoing effect of the intervention. Finally, future research can include qualitative studies to obtain participants' opinions on the optimal time, length, and the number of sessions of dance-based interventions.

## Conclusion

Dance-based interventions have a positive effect on depression outcomes among persons with MCI and dementia. Healthcare providers and dance interventionists in different settings may continue to utilize dance-based interventions for this population. Also, more and higher quality RCT studies with larger sample sizes are recommended to be advocated and implemented. The effects of dance-based interventions on depression and anxiety outcomes should be measured and monitored to investigate how these interventions help persons with MCI and dementia decrease depression and anxiety, and help them keep active coping behaviors, to maintain hope and improve quality of life and resilience.

## Data Availability Statement

The original contributions presented in the study are included in the article/supplementary material, further inquiries can be directed to the corresponding author.

## Author Contributions

YW conceived the study, conducted the meta-analysis, wrote the manuscript, and supervised the overall project. YW, YT, ZD, JW, HC, and DS screened the title, abstract and full text, and extracted data. ML and IC reviewed and proofread the manuscript. All authors contributed to the article and approved the submitted version.

## Conflict of Interest

The authors declare that the research was conducted in the absence of any commercial or financial relationships that could be construed as a potential conflict of interest.

## Publisher's Note

All claims expressed in this article are solely those of the authors and do not necessarily represent those of their affiliated organizations, or those of the publisher, the editors and the reviewers. Any product that may be evaluated in this article, or claim that may be made by its manufacturer, is not guaranteed or endorsed by the publisher.
